# Viral Infections and Autoimmune Disease: Roles of LCMV in Delineating Mechanisms of Immune Tolerance

**DOI:** 10.3390/v11100885

**Published:** 2019-09-21

**Authors:** Georgia Fousteri, Amy Dave Jhatakia

**Affiliations:** 1 Division of Immunology Transplantation and Infectious Diseases (DITID), Diabetes Research Institute (DRI) IRCCS San Raffaele Scientific Institute, 20132 Milan, Italy; 2 Bristol-Myers Squibb, Redwood City, CA 94063, USA; adave323@gmail.com

**Keywords:** lymphocytic choriomeningitis virus (LCMV), viral infection, autoimmunity, molecular mimicry, bystander activation, immune tolerance

## Abstract

Viral infections are a natural part of our existence. They can affect us in many ways that are the result of the interaction between the viral pathogen and our immune system. Most times, the resulting immune response is beneficial for the host. The pathogen is cleared, thus protecting our vital organs with no other consequences. Conversely, the reaction of our immune system against the pathogen can cause organ damage (immunopathology) or lead to autoimmune disease. To date, there are several mechanisms for virus-induced autoimmune disease, including molecular mimicry and bystander activation, in support of the “fertile field” hypothesis (terms defined in our review). In contrast, viral infections have been associated with protection from autoimmunity through mechanisms that include Treg invigoration and immune deviation, in support of the “hygiene hypothesis”, also defined here. Infection with lymphocytic choriomeningitis virus (LCMV) is one of the prototypes showing that the interaction of our immune system with viruses can either accelerate or prevent autoimmunity. Studies using mouse models of LCMV have helped conceive and establish several concepts that we now know and use to explain how viruses can lead to autoimmune activation or induce tolerance. Some of the most important mechanisms established during the course of LCMV infection are described in this short review.

## 1. Introduction

Lymphocytic choriomeningitis virus (LCMV) is a prototype viral system that has been used to address several mechanisms of tissue-specific tolerance. Generally, the LCMV system has been used to understand the mechanisms that induce or break tolerance at a tissue/organ level, causing autoimmune-mediated tissue damage that resembles the clinical features of human autoimmune disease [1]. This system has also been used to address the efficacy of therapeutic strategies to prevent or reverse autoimmune disease progression, as well as the safety of those treatments in the context of a viral infection.

Transgenic mouse models that express viral proteins (model antigens) in specific tissues have allowed for the precise tracking of antiviral/autoimmune responses. Furthermore, crossing to other gene-deficient or transgenic mice has unraveled the molecules that mediate tissue-specific tolerance [2]. T-cell-receptor (TCR) transgenic mouse models that specifically recognize model antigens have been used to trace and characterize the autoreactive T cells after adoptive transfer in vivo. These “reductionist approaches” have allowed investigators to decipher basic mechanisms that control immune activation and determine tolerance [3]. Although these models may not recapitulate all aspects of human disease, they primarily serve to elucidate the role of environmental triggers and viral infections in autoimmune disease pathogenesis [1,4,5]. They can also be used to address how the combination of genetic and environmental factors converges and defines disease susceptibility. In this review, we summarize the concepts that emerged by studying autoimmune disease development in mouse models of autoimmunity using the LCMV system. We also address how LCMV infection has helped unravel the mechanisms by which viral infections promote peripheral tolerance. 

## 2. LCMV-Induced Mouse Models of Autoimmunity

Infection with LCMV can cause viral encephalitis in humans and is principally transmitted by rodents [6,7]. LCMV is not a lytic virus, and is able to generate robust cytotoxic lymphocyte responses. As a consequence, tissue inflammation after LCMV infection is caused by the immune system. Central nervous system (CNS) infection by LCMV in mice leads to an intense antiviral T-cell response and consequent fatal choriomeningitis [8,9]. The development of LCMV-induced meningitis was the first example of disease caused as a collateral immune damage—a process now known as immunopathology (pathology of a tissue, organ system, or disease caused by the immune system). These experiments have demonstrated how infection with a non-cytolytic virus activates the immune system and leads to autoimmunity. Below, we briefly describe some of these LCMV-induced models of autoimmunity and the concepts that were formed through the studies.

**Figure 1:**
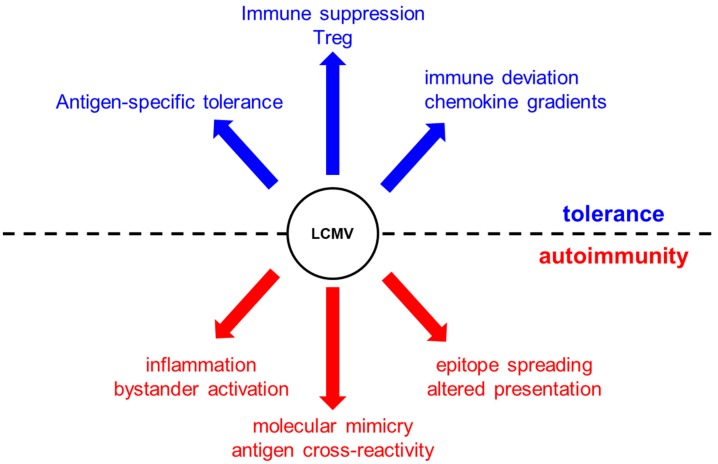
Lymphocytic choriomeningitis virus (LCMV) infection can induce tolerance and promote autoimmunity via mechanisms that include antigen-specific tolerance, immune suppression (death of autoreactive T cells) Treg invigoration, and immune deviation via chemokine gradients (e.g., CXCL10). In contrast, virus infection can initiate or propagate an autoimmune disease via epitope spreading and molecular mimicry, inflammation, and activation of antigen-presenting cells (APCs) that present self-antigens.

In 1991, a breakthrough in our understanding of the role of viruses in triggering autoimmunity came from two studies published in *Cell* describing the LCMV-induced model of autoimmune diabetes [10,11]. Two independent groups led by Zinkernagel and Oldstone showed for the first time that transgenic mice expressing the glycoprotein (GP) or nucleoprotein (NP) of LCMV as a self-antigen in their islets (under the control of the rat insulin promoter, RIP) can develop diabetes after viral clearance between 10 and 15 days after infection with LCMV. RIP-LCMV diabetic mice predominantly developed a T-cell (CD8)-mediated acute form of autoimmune diabetes. Interestingly, autoreactive T cells (and antibodies) were not only specific to LCMV, but also to islet antigens. Thus, a single infection with LCMV led to the breakdown of tolerance to islet antigens through mechanisms known today as molecular mimicry (a phenomenon where sequence similarities between foreign and self-peptides result in the cross-activation of autoreactive T or B cells by pathogen-derived antigens), bystander activation (a phenomenon where T cells specific for an antigen become activated during an immune response against an unrelated antigen), and antigen spreading (a phenomenon in which the immune system expands its response beyond the immunodominant epitopes first recognized by T and B cells; these antigens may be tissue antigens and not necessarily viral epitopes), in support of the fertile field hypothesis (Figure 1).

One mouse model of autoimmune hepatitis has heavily relied on the same concept of viral-induced disease. This model uses a similar approach as the RIP-LCMV model of autoimmune diabetes described above. More precisely, the GP or NP protein of LCMV is expressed in transgenic mice under the control of the albumin promoter (Alb) [12,13,14]. However, in contrast to RIP-LCMV mice, Alb-LCMV mice develop transient hepatitis following infection with LCMV due to the strong tolerogenic nature of the liver. An additional adoptive transfer of GP_33–41_-specific CD8 T cells (named P14) from T-cell receptor (TCR) transgenic mice is required to definitively break tolerance in Alb-LCMV mice and cause long-lasting autoimmune hepatitis. Of note, P14 T-cell transfer in either RIP-LCMV or Alb-LCMV does not cause disease, suggesting that LCMV-induced inflammation is necessary to break tolerance in this setting. This became the basis of another immunological concept known as immune ignorance (a phenomenon where weakly self-recognizing T cells fail to recognize peripheral antigen, and consequently fail to become activated), which is described further below.

A mouse model of experimental autoimmune encephalomyelitis similar to the RIP-LCMV and Alb-LCMV models has also been established. Transgenic mice were generated to express the NP or GP of LCMV in oligodendrocytes under the guidance of the myelin base protein (MBP) promoter [15,16]. Intraperitoneal infection with LCMV in MBP-LCMV mice led to the infection of tissues in the periphery but not the CNS, and the virus was cleared within 7–14 days. After clearance, a chronic inflammation of the CNS occurred, characterized by upregulation of major histocompatibility (MHC) class I and II molecules. A second LCMV infection led to enhanced CNS pathology, characterized by the loss of myelin and clinical motor dysfunction. Disease enhancement also occurred after a second infection with unrelated viruses that cross-activated LCMV-specific memory T cells [15,16]. Similar to the previous models, this model allowed investigators to establish the concepts of molecular mimicry, bystander activation, and antigen spreading as potential mechanisms of autoimmunity triggered by infection.

Through the use of these models of virus-induced autoimmune disease, researchers have been able to investigate several basic mechanisms of immune activation and tolerance and to assess the efficacy and safety of novel therapies. These mouse models also serve to address the immunosuppressive action of LCMV and its role in inhibiting autoimmune disease progression through several mechanisms. Below, we describe the lessons learned and concepts formed from the study of these models and also address how LCMV infection can promote immunological tolerance in different settings.

## 3. Mechanisms That Can Lead to Autoimmunity Following Viral Infection

### 3.1. Clonal Deletion, T-Cell Anergy, and Immune Ignorance

Before the discovery of viral persistence, it was thought that a virus-infected host would either succumb to or clear the infection [17,18]. That is, either the immune system wins and clears the infection, or the infection overcomes the immune system and kills the host. However, early studies showed that this was not the case. Mice infected with LCMV in utero or shortly after birth with a viral dose capable of killing an adult mouse were shown to “tolerate” the infection and survive with high viral titers present in their blood [19,20]. These newborn mice were persistently infected with LCMV because they were immunologically tolerant to the virus.

One of the most common experiments done back in the 1990s was the crossing of TCR transgenic mice to antigen-expressing mice. When TCR (GP_33–41_-specific, P14) transgenic mice were crossed with mice ubiquitously expressing the LCMV GP antigen, the clonal deletion (death of the same TCR-bearing T cells in the thymus due to strong antigen recognition) of T cells was seen in the thymus at the early CD4^+^ CD8^+^ double-positive stage [21]. The remaining cytotoxic T lymphocytes (CTLs) were unresponsive, and virus persisted upon infection. The state of these cells is known as anergy (inability to respond to TCR stimulation). However, when the same TCR transgenic mice were crossed with RIP-LCMV mice where the antigen was expressed on pancreatic beta islet cells, CTL reactivity was normal. These experiments became the basis of another mechanism of peripheral tolerance known as immune ignorance [22]. This term was originally coined by Ohashi and colleagues to describe LCMV-reactive T lymphocytes present in RIP-LCMV mice crossed with LCMV-specific TCR transgenic P14 mice. LCMV-specific T cells were neither deleted nor anergic, but were unaffected by the presence of LCMV antigens on pancreatic beta cells [10]. The adoptive transfer of P14 mice in RIP-LCMV or Alb-LCMV mice did not activate the cells through the same mechanism. This state of tolerance (ignorance) could be overcome upon LCMV infection, demonstrating that the appropriate presentation of the self-epitope on antigen-presenting cells (APCs) promptly induces effector T cells and causes disease (diabetes or hepatitis, respectively). Generally, inflammation caused by infections is thought to be one of the leading mechanisms activating autoreactive T cells [23].

### 3.2. Molecular Mimicry, Epitope Spreading, and Bystander Activation

The hypothesis of molecular mimicry dates several decades back, and is the basis of experimental autoimmune animal models that are used, including the ones we described above. These models serve to support the “fertile field” hypothesis (a hypothesis suggesting that infections favor autoimmune disease development). Molecular mimicry suggests that environmental factors such as viruses potentiate an autoimmune process by activating autoreactive T cells that recognize viral epitopes due to cross-reactivity [16,24,25]. The mechanism of molecular mimicry has been proposed to account for the connection between coxsackievirus B3 (CVB) infection and autoimmune diabetes and myocarditis [26,27,28]. The same mechanism was found responsible for experimental allergic encephalomyelitis in rabbits [29].

Another possible mechanism that could account for the activation of autoreactive T cells by virus infection is bystander activation. This model suggests that autoreactive T cells become “bystander” activated due to viral-induced inflammatory events causing tissue damage and the release of sequestered tissue antigens, leading to an enhanced self-antigen-presenting activity by APCs [30,31,32]. This concept seems to be the case for autoreactive memory T cells, as these cells become more effectively activated than naïve T cells from repeated infections with viruses of unrelated specificity [33,34]. Possibly, both molecular mimicry and bystander activation act in precipitating autoimmunity, as shown in an experimental model of multiple sclerosis [35].

An additional mechanism that contributes to autoimmune disease predisposition is epitope spreading [36]. Today we know that B- and T-cell immune responses are not static but continue to evolve throughout the course of antigenic exposure. This phenomenon contributes to the activation of T cells of additional specificities [37]. The concept of epitope spreading was once again demonstrated using the LCMV viral system. Immune responses to LCMV are different when acute and chronic T-cell epitopes are compared. In the acute response to LCMV, T cells are restricted to two immunodominant peptides (GP_33–41_ and NP_396–404_), in part based on the high affinity of T cells for these peptides. In contrast, chronic T-cell responses that arise and persist long after the clearance of the virus are directed at subdominant determinants with lesser affinity to MHC [38]. In epitope spreading, the response does not always broaden; the magnitude of the responses to the known immunodominant epitopes can change as well [38]. Epitope spreading, especially during chronic infections, could potentially lead to the activation of cross-reactive, low-affinity autoreactive T cells that could fuel the autoreactive process in autoimmunity. Persistent viral infections can lead to immune-mediated injury also due to the constant presence of viral antigen driving the immune response. This mechanism has been extensively studied in the mouse model of CVB-mediated myocarditis, and it is reviewed elsewhere [5,39].

Viral exposure can also lead to responses that are not related to the original pathogen [40,41,42,43], which in the case of transplantation represent a potent barrier of tolerance induction [44]. This phenomenon, termed heterologous immunity (immunity that develops against one pathogen after a host has had exposure to non-identical pathogens), occurs by several mechanisms, including TCR cross-reactivity and non-specific bystander activation [45,46,47,48]. Infection with LCMV at the time of transplantation was shown to inhibit the beneficial effects provided by co-stimulation blockade, thus preventing the establishment of tolerance [46]. Our group has demonstrated that LCMV infection did not break transplant tolerance once it was established after the adoptive transfer of donor-specific T regulatory type 1 (Tr1) cells or treatment with G-CSF/rapamycin [49,50]. Interestingly, analysis of the alloreactive repertoire in LCMV mice showed that LCMV increased the number of donor-specific T cells by a mechanism that remains unclear [46].

### 3.3. T-Cell Exhaustion and Immunopathology

Another important mechanism of T-cell unresponsiveness and the state of tolerance is known as T-cell exhaustion (a state of dysfunctional T cells characterized by progressive loss of function, changes in transcriptional profiles, and sustained expression of inhibitory receptors) [51,52,53]. T cells show a strong expression of co-inhibitory molecules including PD-1, LAG-3, and CTLA-4 during infection with LCMV [54,55,56]. While the expression of these molecules is downregulated in activated virus-specific T cells after the clearance of an acute infection, its expression remains high after infection with viral strains such as LCMV Clone 13, which causes persistent infection [56,57]. This high PD-1 expression by the T cells results in increased interaction with the PD-L1-expressing parenchymal cells of the infected tissues, and is associated the anergic phenotype of T cells [58]. These exhausted T cells show high expression of inhibitory receptors (TIM3, LAG-3, etc.) and poor effector functions. The T-cell response is augmented via the administration of blocking antibodies for PD-1 and other inhibitory receptors in both acute and chronic infection, suggesting that PD-1–PD-1 ligand interaction attenuates T-cell activation [57,59,60]. Currently, checkpoint inhibitors targeting PD-1 and other checkpoint receptors are used in the clinic to counteract the exhausted state of T cells in patients with advanced cancer [61,62]. While blocking checkpoint inhibitors have been successful in the treatment of cancer, immune-related adverse events have been observed, such as inflammatory bowel disease or thyroiditis. This observation indicates a crucial role for the CTLA-4 and PD-1 inhibitory molecules in normal tolerance mechanisms [63].

One obvious question is, why would there be need of a control system to attenuate T-cell activation, thus perturbing viral clearance? It seems that inhibitory molecules such as PD-1 protect the host by preventing a strong T-cell attack against infected cells. This idea is supported by the animal model of chronic infection with LCMV. When PD-L1 knockout mice were infected by LCMV Clone 13, all the mice died of severe immune inflammation due to an exaggerated T-cell response (uncontrolled production of effector cytokines by effector T cells, causing damage to tissue cells and organ destruction) [64]. This exaggerated response causing tissue damage is now known as immunopathology. Thus, PD-1 acts by slowing the course of immune response during infection, limiting a rapid and possibly more aggressive response that could lead to tissue destruction and immunopathology.

While PD-1 restricts T-cell activation to limit immunopathology following an infection, this molecule is essential to promote self-tolerance to autoantigens. Mice deficient for PD-1 develop a late-onset lupus-like autoimmune syndrome on the C57BL/6 and lethal dilated cardiomyopathy on the BALB/c background [65,66]. NOD mice with a null mutation of PD-1 or its ligands show heightened disease penetrance, earlier onset, and more severe diabetes progression than control mice [67,68]. Similarly to NOD, MRL mice that are prone to autoimmunity develop severe myocarditis and pneumonia when they lack either PD-1 or PD-L1, and more than 70% of these mice die within the first 10 weeks of age [69]. Thus, a molecule that was discovered to control T-cell activation and exhaustion in LCMV infection was found to be essential for T-cell tolerance to autoantigens in several disease settings.

## 4. The Hygiene Hypothesis and How Viral Infections Protect from Autoimmunity

Epidemiological data indicate that infections play a role in preventing autoimmunity. The decline in the incidences of infectious diseases and the increase in the frequency of autoimmune diseases suggest that there might be a link between the two phenomena. These observations led to the hygiene hypothesis, which postulates that the increase in the frequency of autoimmune diseases is possibly due to a reduction in the frequency of infections [70]. Although this hypothesis in humans is supported by epidemiological data, experiments with mouse models of LCMV were consistent. Autoimmune disease was prevented by infection [71,72]. Specifically, prediabetic NOD mice infected with LCMV (or with CVB3) were fully protected from developing T1D [72,73,74,75]. Viral infections promote tolerance through several mechanisms, including antigen-specific tolerance (induction of tolerance to specific antigens via exogenous administration), Treg induction/invigoration (an increase in the number or functionality of Treg cells), immune deviation (modification of the immune response to an antigen caused by a previous exposure to the same antigen), many of them discovered and established using the LCMV viral system (Figure 1).

### 4.1. Antigen-Specific Tolerance

As explained above, molecular mimicry and antigen cross-reactivity is one of the mechanisms by which viruses promote autoimmunity. Paradoxically, the same mechanism can promote tolerance and prevent autoimmunity [76]. A cross-reactive epitope is a tolerizing antigen instead of promoting autoimmunity. For instance, when mice (strains PL/J and SJL/J) were infected with a vaccinia virus (VV) encoding for the first 23 immunodominant amino acids of myelin base protein (MBP), these mice did not develop experimental autoimmune encephalomyelitis (EAE) and were protected from the subsequent induction of EAE via MBP peptide immunization [77]. Interestingly, when the infected mice were immunized with whole MBP, tolerance prevailed. However, mice were not protected against EAE when whole spinal cord lysate was used to induce EAE, suggesting that antigen-specific tolerance had occurred. Thus, the protection elicited by VV-recombinant-MBP infection was specific to the MBP antigen and not all encephalitogenic antigens. Peptide-specific tolerance was also established in the RIP-LCMV mouse model of T1D after synthetic (GP) peptide treatment [78].

Interestingly, in EAE experiments, the part of MBP that was cloned in VV was not acetylated as in the native molecule, suggesting that antigen-specific tolerance occurred via the presentation of an altered peptide ligand (APL) [79,80]. One of the possible mechanisms of the induction of tolerance after viral infection is the involvement of APLs as tolerizing antigens. This knowledge has had a significant impact on the way antigen-specific therapies are designed. For some diseases, APLs were shown to be more effective than native epitopes at inducing tolerance [81]. These experiments pointed to applications for APL in antigen-specific therapy to prevent autoimmune disorders [82,83].

### 4.2. Immune Suppression, Treg Invigoration, and Immune Deviation

Infection with CVB or LCMV can abrogate the development of T1D in NOD and RIP-LCMV prediabetic mice when infected early in the disease pathogenesis [73,74,75]. Mechanistically, the attenuation of disease can be explained by the upregulation of PD-L1 and TNF production, as well as the bystander activation of protective Treg cells. More precisely, viral infection induced the expression of PDL-1 on lymphoid cells, which prevented the expansion of diabetogenic CD8^+^ T cells expressing PD-1 and increased the frequency of TGFβ-producing CD4^+^CD25^+^FOXP3^+^ Treg cells [74,75]. Furthermore, adoptive cell transfer of these Tregs into NOD mice protected these mice from developing T1D, suggesting that the viral infection invigorated the Treg function and fitness [75]. Further experiments showed that the enhancing effects of CVB on the NOD Tregs were mainly elicited through toll-like receptor 2 (TLR2) expression [74].

Another interesting observation made by the use of the LCMV viral infection in NOD and RIP-LCMV mice was the following: the infection of prediabetic mice resulted in substantial viral growth in the pancreatic lymph nodes but not the pancreas (or islets). High inflammation due to viral growth resulted in the elevation of CXCL10 levels, specifically in the pancreatic lymph nodes. This elevation led to a change in the migration of the inflammatory (autoreactive) lymphocytes from the islets in the pancreas to the pancreatic lymph nodes. As a result, the number of autoreactive lymphocytes in the islets of prediabetic mice was drastically reduced [73,84]. This mechanism is now known as immune deviation. Interestingly, a significant increase in the apoptosis of antigen-specific autoreactive T cells was observed as a result of the hyperactivation or activation of Tregs. Thus, in addition to immune deviation, immune suppression, hyperactivation, and activation-induced cell death contributed to the protection of mice from autoimmune diabetes [85,86].

## 5. Conclusions

Much of our knowledge about several clinically relevant immune processes is derived from studies in the mouse model of LCMV infection. Studies using this model have formed the foundation for our understanding of human T-cell activation, contraction and memory development, and tolerance. LCMV and transgenic mouse models have been used to evaluate the efficacy of immunotherapies in treating T-cell exhaustion, preventing/reversing autoimmunity, and assessing the safety of these treatments in the context of viral infections. Our understanding of T-cell immunity to pathogens and self-tolerance to autoantigens has developed in the past three decades with the use of LCMV. Given the rich history of using the mouse model of LCMV towards understanding basic immunology, we believe that this system will continue to provide us with clarity on a wide variety of unaddressed questions in immunology, translational research, and beyond.
